# Supplementary material for: A vagal reflex evoked by airway closure
Source: Nature. 2024 Mar 6;627(8005):830–8. doi: 10.1038/s41586-024-07144-2 (PMC10972749; doi:10.1038/s41586-024-07144-2)
Supplement: Supplementary file 1 — Reporting Summary [file 41586_2024_7144_MOESM1_ESM.pdf]

nature portfolio

Corresponding author(s): Stephen Liberles

Last updated by author(s): Dec 19, 2023

Reporting Summary

Nature Portfolio wishes to improve the reproducibility of the work that we publish. This form provides structure for consistency and transparency in reporting. For further information on Nature Portfolio policies, see our Editorial Policies and the Editorial Policy Checklist.

Statistics

For all statistical analyses, confirm that the following items are present in the figure legend, table legend, main text, or Methods section.

n/a	Confirmed	
☐	☒	The exact sample size (*n*) for each experimental group/condition, given as a discrete number and unit of measurement
☐	☒	A statement on whether measurements were taken from distinct samples or whether the same sample was measured repeatedly
☐	☒	The statistical test(s) used AND whether they are one- or two-sided<br>*Only common tests should be described solely by name; describe more complex techniques in the Methods section.*
☒	☐	A description of all covariates tested
☐	☒	A description of any assumptions or corrections, such as tests of normality and adjustment for multiple comparisons
☐	☒	A full description of the statistical parameters including central tendency (e.g. means) or other basic estimates (e.g. regression coefficient) AND variation (e.g. standard deviation) or associated estimates of uncertainty (e.g. confidence intervals)
☐	☒	For null hypothesis testing, the test statistic (e.g. *F*, *t*, *r*) with confidence intervals, effect sizes, degrees of freedom and *P* value noted<br>*Give P values as exact values whenever suitable.*
☒	☐	For Bayesian analysis, information on the choice of priors and Markov chain Monte Carlo settings
☒	☐	For hierarchical and complex designs, identification of the appropriate level for tests and full reporting of outcomes
☒	☐	Estimates of effect sizes (e.g. Cohen's *d*, Pearson's *r*), indicating how they were calculated

Our web collection on statistics for biologists contains articles on many of the points above.

Software and code

Policy information about availability of computer code

Data collection	Biopac AcqKnowledge (v4.2, 4.5, and 5.0), flexiWare v8.2, Olympus FluoView software vFV31S-SW, Leica LAS AF v2.3.6, Prizmatix Pulser v2.3.1, lox2 software SCIREQ v2.10.5.28, Zen Blue software v2.6 and 3.2, ImSpector v7.1.4
Data analysis	Biopac AcqKnowledge (v4.2, 4.5, and 5.0), flexiWare v8.2, lox2 software SCIREQ v2.10.5.28, Fiji ImageJ v1.52p, R (v4.1.3) using Seurat (v4.1.1), Cell Ranger (pipeline v3.1.0) Enrichr (https://maayanlab.cloud/Enrichr/ ; October 1, 2022), Microsoft Excel (Office 365), Prism v9 (GraphPad), BORIS v8.20.4

For manuscripts utilizing custom algorithms or software that are central to the research but not yet described in published literature, software must be made available to editors and reviewers. We strongly encourage code deposition in a community repository (e.g. GitHub). See the Nature Portfolio guidelines for submitting code & software for further information.

Data

Policy information about availability of data

All manuscripts must include a data availability statement. This statement should provide the following information, where applicable:

- Accession codes, unique identifiers, or web links for publicly available datasets
- A description of any restrictions on data availability
- For clinical datasets or third party data, please ensure that the statement adheres to our policy

Source data used for figures are provided, and single-cell transcriptome data of NEBs (NCBI GEO) are publicly available (GEO accession number to be determined).

Research involving human participants, their data, or biological material

Policy information about studies with human participants or human data. See also policy information about sex, gender (identity/presentation), and sexual orientation and race, ethnicity and racism.

Reporting on sex and gender	N/A
Reporting on race, ethnicity, or other socially relevant groupings	N/A
Population characteristics	N/A
Recruitment	N/A
Ethics oversight	N/A

Note that full information on the approval of the study protocol must also be provided in the manuscript.

Field-specific reporting

Please select the one below that is the best fit for your research. If you are not sure, read the appropriate sections before making your selection.

☒ Life sciences ☐ Behavioural & social sciences ☐ Ecological, evolutionary & environmental sciences

For a reference copy of the document with all sections, see nature.com/documents/nr-reporting-summary-flat.pdf

Life sciences study design

All studies must disclose on these points even when the disclosure is negative.

Sample size	Sample sizes were determined based on previous expertise and publications in our field (for example, PMID: 26855425, 28360327, 28002412, 36750092). Exact sample sizes are described in each figure legend or Methods.
Data exclusions	For behavioral analysis in Extended Data Fig. 7, occasional animals (3/24) tested were excluded because the animal was oriented away from the camera for most of the session, hindering accurate behavioral scoring. Exclusions were determined by a genotype-blind investigator.
Replication	All replicates were biological, unless otherwise indicated. All figures depicting representative images were independently replicated at least twice, but typically three or more times, and details are described in figure legends.
Randomization	Animals were randomly assigned to experimental cohorts, based on genotyping and age-matching.
Blinding	Investigators were genotype-blind to group allocations for plethysmography, physiological experiments, and behavioral analysis associated with Figures 3, 4, 5, and Extended Data Fig. 7. Blinding for experiments involving nerve transections, optogenetics, and comparisons of airway stimuli within a cohort was not possible as the same investigator applied the perturbation and recorded the response, and the perturbations are necessarily apparent in recordings for analysis.

Reporting for specific materials, systems and methods

We require information from authors about some types of materials, experimental systems and methods used in many studies. Here, indicate whether each material, system or method listed is relevant to your study. If you are not sure if a list item applies to your research, read the appropriate section before selecting a response.

Materials & experimental systems

n/a	Involved in the study	
☐	☒	Antibodies
☒	☐	Eukaryotic cell lines
☒	☐	Palaeontology and archaeology
☐	☒	Animals and other organisms
☒	☐	Clinical data
☒	☐	Dual use research of concern
☒	☐	Plants

Methods

n/a	Involved in the study	
☒	☐	ChIP-seq
☐	☒	Flow cytometry
☒	☐	MRI-based neuroimaging

Antibodies

Antibodies used	Primary Antibodies: anti-NCAM1, 1:250, Cell Signaling Technology, 99746S; anti-GFP, 5 microgram/ml, Aves Labs, GFP-1020; anti-mCherry/RFP, 3 microgram/ml, OriGene Technologies, AB0040-200; anti-HB-EGF (human), 1:250, R&D Systems, AF-259-NA; anti-RFP, Rockland, 1:1000, Rockland, 600-401-379.<br><br>Secondary Antibodies: Jackson Immunoresearch: anti-Chicken IgG-Alexa fluor 488, anti-rabbit IgG-Cy3, anti-rabbit IgG Cy5, anti-goat IgG Cy5, anti-goat IgG Cy3; Secondary antibody catalog numbers are RRIDs AB_2340375, AB_2307443, AB_2340607, AB_2340415, AB_2307351, respectively
Validation	Primary and secondary antibodies are commercially available and validated by the manufacturers. In our previous work with anti-DTR, GFP, and RFP antibodies, background staining was not observed in wild type animals lacking antigen (PMID: 31747594, 32259485, 33278342, 36890237)<br><br>Manufacturer Validation and Quality Control Practices:<br><br>Cell Signaling Technologies: https://www.cellsignal.com/about-us/cst-antibody-validation-principles<br><br>Aves Labs: "Antibodies were analyzed by western blot analysis (1:5000 dilution) and immunohistochemistry (1:500 dilution) using transgenic mice expressing the GFP gene product. Western blots were performed using BlokHen® (Aves Labs) as the blocking reagent, and HRP-labeled goat anti-chicken antibodies (Aves Labs, Cat. #H-1004) as the detection reagent"<br>OriGene Technologies: "In 293HEK cells transfected with cds plasmid detects a band of 29 kDa by Western blot. This antibody (AB0040) recognizes very well tdTomato and does not recognize GFP (green fluorescent protein)"<br><br>R&D Systems: "Detects human HB-EGF in ELISAs and Western blots. In direct ELISAs, less than 1% cross reactivity with recombinant mouse HB-EGF is observed. In sandwich immunoassays, less than 0.1% cross-reactivity with recombinant human (rh) Amphiregulin, rhBetacellulin, rhEpiregulin, and recombinant mouse Epigen is observed. "<br><br>Rockland: "This product was prepared from monospecific antiserum by immunoaffinity chromatography using Red Fluorescent Protein (Discosoma) coupled to agarose beads followed by solid phase adsorption(s) to remove any unwanted reactivities. Expect reactivity against RFP and its variants: mCherry, tdTomato, mBanana, mOrange, mPlum, mOrange and mStrawberry. Assay by immunoelectrophoresis resulted in a single precipitin arc against anti-Rabbit Serum and purified and partially purified Red Fluorescent Protein (Discosoma). No reaction was observed against Human, Mouse or Rat serum proteins."<br><br>Jackson Immunoresearch: Based on immunoelectrophoresis and/or ELISA, the antibody reacts with whole molecule of host Ig. It also reacts with the light chains of other host species immunoglobulins. No antibody was detected against non-immunoglobulin serum proteins. The antibody has been tested by ELISA and/or solid-phase adsorbed to ensure minimal cross-reaction with non-host species such as chicken, guinea pig, syrian hamster, goat, horse, human, mouse, rabbit and rat serum proteins, but it may cross-react with immunoglobulins from other species. Whole IgG antibodies are isolated as intact molecules from antisera by immunoaffinity chromatography. They have an Fc portion and two antigen binding Fab portions joined together by disulfide bonds and therefore they are divalent. The average molecular weight is reported to be about 160 kDa. The whole IgG form of antibodies is suitable for the majority of immunodetection procedures and is the most cost effective.

Animals and other research organisms

Policy information about studies involving animals; ARRIVE guidelines recommended for reporting animal research, and Sex and Gender in Research

Laboratory animals	Animals were maintained under constant environmental conditions (23±1 degree C, 46±5% relative humidity) with food and water provided ad libitum in a 12-h light-dark cycle. All studies used adult male and female mice in comparable numbers from mixed genetic backgrounds and ages 6 to 24 weeks old. All CreER mice and control littermates received tamoxifen (Sigma T5648, 100 mg/kg, IP, sunflower oil, twice 48 hrs apart) at least 10 days prior to further experiments. Mice containing Cre- and Flp-dependent DTR alleles were a generous gift from Martyn Goulding, and Calca-egfp mice were purchased (GENSAT, RRID:MMRRC_011187-UCD). For Pvalb-t2a-Cre, only female Cre mice were used for husbandry due to reported germline recombination in male breeders (Jax, 012358); male and female offspring were used for experiments. Olfr78-p2a-Cre mice were generated by pronuclear injection of Cas9 protein, CRISPR sgRNAs targeting the Olfr78 locus 3' UTR, and a single strand DNA template containing a p2a-cre gene cassette with 150 bp homology arms into C57BL/6 embryos. Knock-in pups were screened by PCR analysis, and correct expression of the transgene was verified by RNA in situ hybridization. All Cre driver lines used are viable and fertile, and abnormal phenotypes were not detected. All other mice were purchased from Jackson Laboratory, or made in the lab and then deposited at Jackson Laboratory: Ascl1-CreERT2 (012882), Nkx2.1-ires-Flp (028577), Piezo2-egfp-ires-Cre (027719), inter-G-alpha-q-DREADD (26942), lsl-SALSA (31968), lsl-TdTomato (007914), snap25-Gcamp6s (25111), lsl-ChR2 (012569), C57BL/6J (000664), lsl-G-alpha-q-DREADD (026220), loxP-Piezo2 (027720), loxP-Piezo1 (029213), Vglut2-ires2-Flpo (030212), inter-Ai65 (021875), Vglut2-ires-Cre (016963), Npy1r-gfp-Cre (030544), P2ry1-ires-Cre (29284), Pvalb-t2a-Cre (012358), Crhr2-ires-Cre (33728), Npy2r-ires-Cre (29285), Calb1-ires2-Cre (28532), Phox2b-Cre (16223), Glp1r-ires-Cre (29283), Mc4r-2a-Cre (030759), Gpr65-ires-Cre (029282).
Wild animals	No wild animals were used
Reporting on sex	All studies used adult male and female mice in comparable numbers from mixed genetic backgrounds.
Field-collected samples	No field-collected samples were used.

Ethics oversight	All animal procedures followed ethical guidelines outlined in the NIH Guide for the Care and Use of Laboratory Animals, and all procedures were approved by the Institutional Animal Care and Use Committee at Harvard Medical School.

Note that full information on the approval of the study protocol must also be provided in the manuscript.

Flow Cytometry

Plots

Confirm that:

- [ ] The axis labels state the marker and fluorochrome used (e.g. CD4-FITC).
- [ ] The axis scales are clearly visible. Include numbers along axes only for bottom left plot of group (a 'group' is an analysis of identical markers).
- [ ] All plots are contour plots with outliers or pseudocolor plots.
- [ ] A numerical value for number of cells or percentage (with statistics) is provided.

Methodology

Sample preparation	The sample preparation was performed based on previously described tissue dissociations for neuroendocrine and rare epithelial cells (PMID: 30069046, 30069044, 31585080, 36810133, 36469459). In brief, whole lungs below the trachea were harvested from 10 Calca-EGFP and 10 Ascl1-CreER; lsl-tdTomato mice (5-7 weeks old, equal male/female, 10 days after tamoxifen injection), pooled by strain, minced and incubated (60 min, 37°C) in oxygenated papain dissociation buffer (Worthington Biochemical LK003150). Residual tissue was mechanically dissociated through a 100 micron cell strainer, pelleted by centrifugation (400g, 7 min, 4C), washed, resuspended in red blood cell lysis buffer (150 mM NH4Cl, 10 mM NaCHO3, 0.1 mM EDTA) for 5 min, pelleted, and resuspended in FACS buffer (0.5% bovine serum albumin, 2 mM EDTA, PBS, 4C). Immune cells were depleted with anti-CD45 magnetic beads according to the manufacturer's instructions (Biolegend 480027), and the remaining cells were resuspended in viability buffer (TO-PRO-3 and CellTrace Violet, both 1:10,000, in RPMI 1640; Thermo Fisher, T3605, 65-0854-39, 11835030, respectively) prior to sorting by the Immunology Flow Cytometry Core facility (HMS).
Instrument	FACS Aria II (BD Bioscience)
Software	BD FACSDiva Software
Cell population abundance	Abundance of sorted cells were determined by hemocytometer after sorting for 10X Genomics scRNA-seq. Cell population abundance was subsequently determined by scRNA-seq analysis, as depicted in Fig. 5, Extended Data 9 and described in Methods.
Gating strategy	The gating strategy was performed as previously described in exemplary gating plots for isolating neuroendocrine and rare epithelial cells (PMID: 30069046, 30069044, 31585080, 36810133, 36469459). In brief, all events were gated by morphology and complexity (FSC-A vs. SSC-A) to avoid cellular debris, and singlets identified by scatter height-width discrimination (FSC-H vs FSC-W). For sorting gates, unstained, single-stain, and fluorescence minus one controls were used to determine positive and negative boundaries. Live cells were gated on exclusion of TOPRO3 (TOPRO3-negative) and inclusion of CellTrace Violet (CellTrace Violet-positive) prior to gating of fluorescence marker positive cells (RFP+ for Ascl1-CreER; Ai14 and GFP+ for Calca-Egfp, respectively) for sorting.

- [ ] Tick this box to confirm that a figure exemplifying the gating strategy is provided in the Supplementary Information.
